# Measuring cardiac efficiency using PET/MRI

**DOI:** 10.1186/2197-7364-2-S1-A59

**Published:** 2015-05-18

**Authors:** Grand Gullberg, Carina Mari Aparici, Gabriel Brooks, Jing Liu, Julius Guccione, David Saloner, Adam Youngho Seo, Karen Gomes Ordovas

**Affiliations:** Lawrence Berkeley National Laboratory, USA; University of California San Francisco, USA

Heart failure (HF) is a complex syndrome that is projected by the American Heart Association to cost $160 billion by 2030. In HF, significant metabolic changes and structural remodeling lead to reduced cardiac efficiency. A normal heart is approximately 20-25% efficient measured by the ratio of work to oxygen utilization (1 ml oxygen = 21 joules). The heart requires rapid production of ATP where there is complete turnover of ATP every 10 seconds with 90% of ATP produced by mitochondrial oxidative metabolism requiring substrates of approximately 30% glucose and 65% fatty acids. In our preclinical PET/MRI studies in normal rats, we showed a negative correlation between work and the influx rate constant for 18FDG, confirming that glucose is not the preferred substrate at rest. However, even though fatty acid provides 9 kcal/gram compared to 4 kcal/gram for glucose, in HF the preferred energy source is glucose. PET/MRI offers the potential to study this maladapted mechanism of metabolism by measuring work in a region of myocardial tissue simultaneously with the measure of oxygen utilization, glucose, and fatty acid metabolism and to study cardiac efficiency in the etiology of and therapies for HF. MRI is used to measure strain and a finite element mechanical model using pressure measurements is used to estimate myofiber stress. The integral of strain times stress provides a measure of work which divided by energy utilization, estimated by the production of 11CO2 from intravenous injection of 11C-acetate, provides a measure of cardiac efficiency. Our project involves translating our preclinical research to the clinical application of measuring cardiac efficiency in patients. Using PET/MRI to develop technologies for studying myocardial efficiency in patients, provides an opportunity to relate cardiac work of specific tissue regions to metabolic substrates, and measure the heterogeneity of LV efficiency.

